# National Disability Insurance Scheme and Lived Experience of People Presenting to the Emergency Department: Protocol for a Mixed Methods Study

**DOI:** 10.2196/33268

**Published:** 2021-11-04

**Authors:** Heather McIntyre, Mark Loughhead, Laura Hayes, Nicholas Gerard Procter

**Affiliations:** 1 Mental Health and Suicide Prevention Research and Education Group University of South Australia Adelaide Australia; 2 MIND Australia Melbourne Australia

**Keywords:** lived experience, National Disability Insurance Scheme, emergency department, psychosocial disability, communication pathways

## Abstract

**Background:**

Currently, within Australia, 3.6% of all emergency department (ED) presentations are mental health–related. Information about the context of the person presenting to the ED (beyond immediate needs), including their psychosocial disability (PSD) National Disability Insurance Scheme (NDIS) plan, is reported as incomplete and fragmented. There are missed opportunities for early support and care continuity that could potentially inform ED practitioners to revise current practices.

**Objective:**

The aims of this study are: (1) to obtain original data from the lived experience voice of those with the PSD NDIS plan and their experience when presenting to an ED, (2) to gather information from NDIS service providers to reveal communication pathways between the ED and NDIS services, and (3) to gain knowledge from ED clinicians around processes for improving continuity of care and consumer experience.

**Methods:**

This inductive, mixed methods phenomenological study will involve data collection analyzed sequentially, with each stage informing future stages of the research. Interviews will focus on the lived experience voice exploring concerns that have led to an ED presentation, alongside an analysis of associated clinical and administrative documentation and communications. Focus groups with NDIS support workers and support coordinators will provide phenomenological data around the experience from their perspective. National quantitative surveys among those with a PSD NDIS plan and emergency services clinicians will provide insight into current practices within community care and ED presentations. The research project design includes a lived experience advisory group who are assisting with the design of the interview and focus group schedules and national surveys, as well as in shaping the interpretation of qualitative information. All transcripts will be subject to thematic analysis to understand individuals’ meaning-making of these complex and particular phenomena. The research team includes a lived experience researcher and a lived experience carer (PhD candidate).

**Results:**

This study is funded by MIND Australia as a PhD industry scholarship, which commenced in April 2020. A systematic review as a preresearch activity has been completed and is currently under review. The Human Research Ethics Committee of the University of South Australia has approved this project. An advisory group has been selected, and interview, focus group, and survey schedules are currently being codesigned. Recruitment will commence in November 2021. It is envisaged that data collection will be completed by June 2022.

**Conclusions:**

Understanding the lived experience of the precare, during care, and postcare stages of ED presentations from the perspective of those with a PSD NDIS plan will inform the research team around current practices and provide information about improvement for pathways of care for consumers and carers, while also informing health policy.

**International Registered Report Identifier (IRRID):**

PRR1-10.2196/33268

## Introduction

In 2018-2019, there were 8.4 million presentations to public hospital emergency departments (EDs) in Australia, with an average of 23,000 people visiting the ED every day [1,2]. Of these, 303,340 (3.6%) presentations to the EDs in Australia were related to concerns around mental health [2]. In South Australia, there were 519,607 total presentations at EDs [1], with 23,739 (4.5%) of peoples’ presentations classified due to mental health–related distress which represents a 1% increase compared with the national average [2]. People with mental health presentations to the ED can have many challenges cooccurring that have led to an acute crisis. These can include health issues such as diabetes [3]; mental health comorbidity; and/or the complexity of a psychosocial disability (PSD) such as housing instability [4-7], relationship breakdown, substance use [8], disconnection with support networks, or difficulties navigating access across multiple services [9]. Those living with a PSD are among the most disadvantaged in the community [10], as highlighted in the World Health Organization QualityRights report: “Ironically, some of the worst human rights violations and discrimination experienced by people with mental disabilities, intellectual disabilities and substance abuse problems are in health-care settings” [11].

People experiencing mental health crisis periodically have the longest wait times in the ED and, at times, leave before care is completed [12]. Alternatively, consumers can present to the ED repeatedly over several days [13]. South Australia has the longest delay in the country for those presenting to the ED with mental health concerns (16.5 hours compared to the national average of 11.5 hours) [12]. Another element of complexity is that those presenting to EDs may be discharged without follow-up care arranged [14,15]. Postdischarge from clinical care represents a time of greater risk of dying by suicide [16,17], reflecting critical concerns about the quality of support and care being offered [18]. Generally, continuity of care recognizes that consumers have relational continuity with providers who are trusted, provide personalized responses, and have shared understanding of the person’s goals [19].

Lack of psychiatric beds in hospitals can also be a cause for delayed care. In 1998, there were 2943 psychiatric beds in hospitals within Australia, and this number was reduced to 2186 in 2017 [20]. The Organisation for Economic Cooperation and Development reports that the average per country for psychiatric beds is 71 per 100,000 individuals, whereas Australia has only 41 psychiatric beds per 100,000 [21] and South Australia has even less at 30 per 100,000 [9]. As the numbers of psychiatric beds are reducing and consumers are being increasingly discharged from the ED to home (to be supported by community services), the aim of this study is to discover how strategies used for implementing communication pathways contribute to continuity of care and improved experience.

In reviewing the evidence of strengthening discharge communication pathways from the ED to enhance continuity of care, this work can help to improve connection with community mental health services and person-centered, or -led outcomes [22,23]. This research project will obtain data from three sources via three research collection methods and then synthesize and triangulate the data.

## Methods

### Aims

The purpose of this inductive exploratory research study is to discover, describe, and interpret the perspectives of various population groups regarding ED mental health care. Within the context of ongoing disability reform (actively pursued in Australia since the early 1970s), the construction of the National Disability Insurance Scheme (NDIS) was created and called for a “coordinated national approach to improving the delivery of disability services” [24]. The NDIS has been in existence for 8 years with the initial pilot beginning in 2013 [25]. PSD was soon added to the NDIS, with streamlined access for people with a PSD implemented in April 2019 [5,6]. A primary focus of this study will be to understand and clarify the preferred communication pathways between the NDIS, ED, and consumers with a PSD NDIS plan. To inform health policy, this study will discover and interpret the meaning individuals make of their experiences, with an interpretive, mainly qualitative, approach to understanding the life world and meaning-making of participants with lived experience, their carers, NDIS providers, and ED clinicians. To inform the study, a systematic review has been completed by the research team, which has been submitted to a journal and is currently under review, addressing the following research question: What evaluated strategies are used for enhancing clinical outcomes with communication pathways for continuity of care between the emergency department and mental health community support services for people with mental health concerns or who are in suicidal crisis? (see Multimedia Appendix 1 for a description of the search strategy).

### Research Questions

This study focuses on the following emerging research questions based on reporting; gaps in the literature; concerns about the use of isolation and restraint; reports of current practices within the ED; along with consultations with people with lived experience, MIND Australia staff, the research team, and others in the sector:

1. How do those with lived experience, carers, and families experience service integration and coordination across emergency care and their NDIS providers? Are there concerns and preferences that 1. NDIS providers should be alert to prior to clients presenting to the ED? Can awareness of these concerns and preferences be a catalyst to prevent an ED presentation?
2. What are the barriers to accessing therapeutic treatment within the ED through the health/disability/mental health interface (NDIS services and EDs) and how can these be transcended for improved person-centered care and recovery?
3. How do emergency care clinicians connect with the network of NDIS providers in terms of coordination of information; support; and involvement in assessment, treatment planning, and transfer of care? What works well? What does not work well? What could be done better?

### Study Design

**Figure 1:**
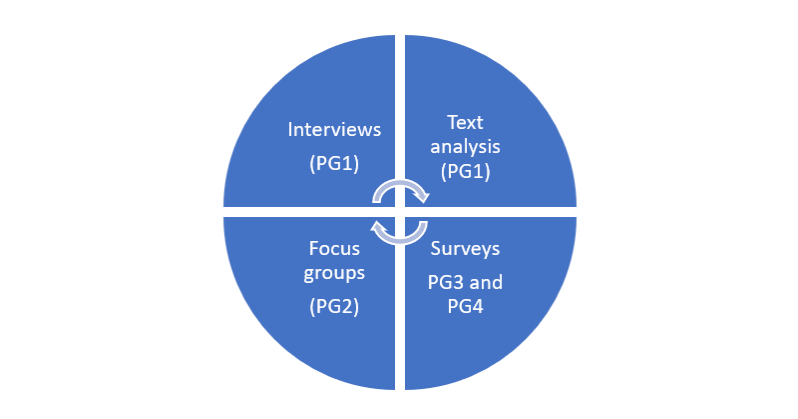
Exploratory research design. PG: participant group.

The study is focused on exploring and interpreting (1) the lived experience of those with a PSD NDIS plan along with an analysis of clinical documentation (participant group 1); (2) the experience of NDIS support coordinator/workers working with people who have a PSD NDIS plan and how they interact with the ED, including communication pathways (participant group 2); (3) the national experience of those with a PSD NDIS plan who present at an ED (participant group 3); and (4) the experience of clinicians in the ED working with people who have a PSD NDIS plan, and how they interact with the NDIS, including communication pathways (participant group 4) (see Figure 1).

Data collection will first aim to elicit the lived experience voice of those with a PSD NDIS plan and carers using semistructured one-to-one interviews (participant group 1). In addition, documents (clinician letters, guides, NDIS plans) and other artifacts (eg, electronic messaging) provided to the person requiring care and/or their carer will also be reviewed to evaluate clinician communication [26]. The phenomenological understanding will continue with NDIS support workers and support coordinators, who will also be recruited to participate in focus groups to explore the current understanding and practice of this population group (participant group 2). Finally, the study will collect national data and understanding from those with a PSD NDIS plan and ED clinicians (participant groups 3 and 4) via an online quantitative survey (see Multimedia Appendix 2).

This exploratory study will enable the discovery of paradoxes and contradictions [27] between three population groups with four different research approaches (interviews, analysis of medical correspondence, focus groups, and quantitative surveys) to compare and contrast themes [28]. An interpretive qualitative approach is considered appropriate for the first two population groups to provide data with rich depth. Primarily, lived experience perspectives, including both consumer and carer perspectives will be generated via interviews and NDIS support workers/coordinators (participant group 2) with focus groups. National quantitative online surveys will be offered to those with a PSD NDIS plan and clinicians working in EDs to give them the flexibility to participate in their own time. The different approaches to the research questions will enable triangulation of data via a convergence of sight lines to bring depth to the analysis.

### Trauma-Informed Approach

This research will be guided by trauma-informed practice principles [29], as many people living with a mental health diagnosis are likely to have experienced significant trauma at some point in their lives, most often during childhood. This will include seeking to avoid retraumatization of participants by focusing on their knowledge about service improvement rather than requiring them to disclose a narrative history of an unpleasant experience in the ED. Participants will be provided with a safe environment (physical or digital) and will be encouraged to bring a support person if that is something they would like to do. To engender trust, the participants will be empowered to cease the interview at any time and to not disclose anything that will cause them distress [30].

The principles behind the 5th National Mental Health and Suicide Prevention Plan [31] and the South Australian Mental Health Strategic Plan [32] clearly underpin the need for the lived experience voice to drive change. This study will align with that principle and be active in incorporating the voices of those with lived experience (ie, those with a PSD, the advisory group, and carers) alongside the mental health workforce. By engaging with a lived experience advisory group, the research team will incorporate their specific expertise to design the interview and focus group schedules and the survey questions.

### Recruitment

Recruitment strategies will be developed in consultation with the advisory group.

Those with lived experience and carers (participant group 1, approximately n=20: lived experience, n=14, carers, n=7; participant group 2, n<20; participant groups 3 and 4, n>50) will be recruited for one-to-one audio-recorded interviews. Potential participants will be given information and opportunity to ask further questions from the research team and will be asked to discuss being involved in the research with their partner, carer, clinician, or friend. Table 1 summarizes the characteristics of each group and the inclusion/exclusion criteria.

**Table 1:** Characteristics and inclusion/exclusion criteria for each participant group.

Participant group	Sample and sample size	Inclusion criteria	Exclusion criteria
1	>20 (lived experience: n=14; carers: n=7)	Aged >18 years; have lived experience and a psychosocial disability NDIS^a^ plan; not in acute care at the time of the interview; presented to the ED^b^ while on a psychosocial disability NDIS plan within the last 2 years; or a carer who will have presented to the ED with a person who has a psychosocial disability NDIS plan	Anyone that is unable to give informed consent; anyone that does not have a psychosocial disability NDIS plan; anyone that has not presented to the ED
2	>20 NDIS support workers	Aged >18 years; NDIS support workers and support coordinators with experience of intervention with people who have presented to the ED	Anyone who is not an NDIS support coordinator or support worker
3	>50 individuals on a psychosocial disability NDIS plan	Same as participant group 1	Same as participant group 1
4	>50 clinicians (including doctors, nurses, psychiatrists, social workers)	Aged >18 years; clinicians (including doctors, nurses, psychiatrists, social workers) who have attended to those presenting at the ED with a psychosocial disability in the last 2 years	Clinicians (including doctors, nurses, psychiatrists, social workers) who have not worked in the ED within the last 2 years

^a^NDIS: National Disability Insurance Scheme.

^b^ED: emergency department.

### Data Analysis

Audio recordings from interviews and focus group (participant groups 1 and 2) will be transcribed and thematically analyzed [28] using NVivo software. Themes will be coded and generated by two members of the research team. Quantitative data (participant groups 3 and 4) will be collated and analyzed using SPSS software. 

## Results

The results of this study will provide insight for strengthening discharge communication pathways between EDs and community mental health services so that these reflect person-centered and person-led care outcomes [22,23]. Meetings have commenced with the advisory group to codesign interview and focus group schedules, along with codesigning the survey questions. Recruitment for participant group 1 will commence imminently. It is envisaged that data collection for participant groups 1 and 2 will be finalized by February 2022. Data collection for the surveys of participant groups 3 and 4 will be completed by mid-2022. This study was approved by the Human Research Ethics Committee of the University of South Australia (application ID 203626) on April 10, 2021.

## Discussion

### Strengths and Limitations

A strength of this study is the inclusion of a lived experienced researcher and a lived experienced carer researcher (PhD candidate) on the research team. The involvement of a lived experience advisory group for interview, focus group, and survey schedule design is another strength of this project. The advisory group will be invited to contribute and be named as authors to the academic papers that will be generated through this research. This will acknowledge their contribution to the design of interview, focus group, and survey questions, and generation of themes from the data. Another strength is that participant groups 1 and 3 are participants with lived experience.

Limitations include that the codesign focuses on authentically shared power but is restricted to the context of a PhD training journey. As this project is a PhD scholarship, true codesign with a lived experience advisory group cannot occur due to the nature of the project and that the PhD candidate is required to demonstrate research skills rather than others doing the work. Nevertheless, aspects of codesign will be included through consultation with a lived experienced advisory group.

### Practical Significance

The NDIS has been in operation for 8 years. Although PSD was included in the initial architecture of the NDIS [33], many of the fundamental design features of the scheme were developed without reference to the needs of this population [34-37]. This research project will be the first of its kind in the Australian context to provide data from the lived experience voice from those with a PSD NDIS plan from the perspective of presenting to the ED in a crisis. The results of this project will inform ED clinicians and NDIS service providers of clearer needs of this population group, along with guiding pathways for better continuity of care.

### Conclusion

This mixed methods study will triangulate the data from interviews among those with lived experience, clinical communications, focus groups with NDIS support workers, and national quantitative surveys among people with a PSD NDIS plan and ED clinicians. This inductive exploratory research study—with an interpretive, qualitative approach—will discover, explore, and describe the lived experience of those with a PSD NDIS plan when presenting to the ED, in retrospect, primarily from the voices of those with lived experience [37].

System encounters, system experiences, and system-wide processes between the hybrid environment of the ED and NDIS services will be explored with the goal of describing this experience and identifying better communication pathways between the various services to enable those with lived experience to have improved voice and influence health policy.

## Appendix

Multimedia Appendix 1 Search strategy for the systematic review (submitted, under review).

Multimedia Appendix 2 Phases of research, population groups, and interventions.

Multimedia Appendix 3 Peer-review report 1 by Clinical & Health Sciences - University of South Australia.

Multimedia Appendix 4 Peer-review report 2 by Clinical & Health Sciences - University of South Australia.

## Abbreviations

ED: emergency department
NDIS: National Disability Insurance Scheme
PSD: psychosocial disability
