# Correction: National Disability Insurance Scheme and Lived Experience of People Presenting to the Emergency Department: Protocol for a Mixed Methods Study

**DOI:** 10.2196/34866

**Published:** 2021-11-25

**Authors:** Heather McIntyre, Mark Loughhead, Laura Hayes, Nicholas Gerard Procter

**Affiliations:** 1 Mental Health and Suicide Prevention Research and Education Group University of South Australia Adelaide Australia; 2 MIND Australia Melbourne Australia

**Keywords:** Lived experience, National Disability Insurance Scheme, emergency department, psychosocial disability, communication pathways

In “National Disability Insurance Scheme and Lived Experience of People Presenting to the Emergency Department: Protocol for a Mixed Methods Study” (JMIR Res Protoc 2021;10(11):e33268) the authors noted some errors. The following changes have been made to correct these errors:

**Title**

In the originally published article, the title read as follows:

National Disability Insurance Scheme and the Lived Experience of Psychosocial Disability for People Presenting to the Emergency Department: Protocol for a Mixed Methods Study

In the corrected version, the title has been revised to:

National Disability Insurance Scheme and Lived Experience of People Presenting to the Emergency Department: Protocol for a Mixed Methods Study

**Author metadata**

In the originally published article, degrees for author Nicholas Gerard Procter appeared as follows:

BASoc, PsycNurs, MBA, PhD

In the corrected version, these degrees are revised as follows:

BA, MBA, PhD

**Corresponding author's address**

In the originally published article, the corresponding address was as follows:

GPO Box 2471

North Terrace

Adelaide, 5001

Australia

Phone: 61 8 8302 1132

Fax: 61 8 8302 2168

In the corrected version, the address has been revised as follows:

School of Nursing and Midwifery

PO Box 2471

Adelaide, 5001

Australia

Phone: 61 419856683

**Abstract**

- Under *Background*, the sentence “There are missed opportunities for early intervention and care continuity that could potentially inform ED practitioners to revise current practices” was changed to “There are missed opportunities for early support and care continuity that could potentially inform ED practitioners to revise current practices.”
- Under *Objective*, the sentence “…to gain knowledge from ED clinicians around processes for continuity of care with this population group” was changed to “…to gain knowledge from ED clinicians around processes for improving continuity of care and consumer experience.”
- Under *Methods*, the sentence “Interviews will focus on the lived experience voice exploring potential indicators that have led to an ED presentation, alongside an analysis of associated clinical and administrative documentation and communications” was changed to “Interviews will focus on the lived experience voice exploring concerns that have led to an ED presentation, alongside an analysis of associated clinical and administrative documentation and communications.”
- Under *Conclusions*, the sentence “…pathways of care for this vulnerable group of people, while also informing health policy” was changed to “…pathways of care for consumers and carers, while also informing health policy.”

**Introduction**

- In the first paragraph, the sentence “In South Australia, there were 519,607 total presentations at EDs, with 23,739 (4.5%) presentations classified as “Mental and Behavioural Disorders,” which represents a 1% increase compared with the national average” was changed to “In South Australia, there were 519,607 total presentations at EDs, with 23,739 (4.5%) of peoples’ presentations classified due to mental health–related distress which represents a 1% increase compared with the national average.”
- In the first paragraph, the sentence “…disconnection with support networks, or the inability to navigate access across multiple services. Those with a PSD…” was changed to “…disconnection with support networks, or difficulties navigating access across multiple services. Those living with a PSD….”
- In the second paragraph, the sentence “Those in a mental health crisis periodically have the longest wait times in the ED and, at times, leave before treatment is completed. Alternatively, they could present to the ED…” was changed to “People experiencing mental health crisis periodically have the longest wait times in the ED and, at times, leave before care is completed. Alternatively, consumers can present to the ED….”
- In the second paragraph, the sentence “Postdischarge from clinical care represents a time of greater risk of dying by suicide, possibly due to inadequate or a rationing of care. Conversely, positive experiences of continuity of care practices contribute to favorable patient outcomes” was changed to “Postdischarge from clinical care represents a time of greater risk of dying by suicide, reflecting critical concerns about the quality of support and care being offered. Generally, continuity of care recognizes that consumers have relational continuity with providers who are trusted, provide personalized responses, and have shared understanding of the person’s goals.”
- In the third paragraph, the sentence “Lack of psychiatric beds in hospitals can also be a cause for delayed treatment” was changed to “Lack of psychiatric beds in hospitals can also be a cause for delayed care.”
- In the third paragraph, the sentence “As the numbers of psychiatric beds are reducing and patients are being increasingly discharged from the ED to home (to be cared for by community services), the aim of this study is to discover how strategies used for implementing communication pathways contribute to continuity of care for this population group” was changed to “As the numbers of psychiatric beds are reducing and consumers are being increasingly discharged from the ED to home (to be supported by community services), the aim of this study is to discover how strategies used for implementing communication pathways contribute to continuity of care and improved experience.”
- In the fourth paragraph, the sentence “…services and outcomes for patients” was changed to “…services and person-centered, or -led outcomes.”

**Methods**

- Under *Aims*, the sentence “PSD was soon added to the NDIS, with streamlined access for those with a PSD implemented in April 2019. A primary focus of this study will be to understand and clarify the preferred communication pathways between the NDIS, ED, and those with a PSD NDIS plan. To inform health policy, this study will discover and interpret human behavior, perceptions, and the meaning individuals make of their experiences…” was changed to “PSD was soon added to the NDIS, with streamlined access for people with a PSD implemented in April 2019. A primary focus of this study will be to understand and clarify the preferred communication pathways between the NDIS, ED, and consumers with a PSD NDIS plan. To inform health policy, this study will discover and interpret the meaning individuals make of their experiences….”
- Under *Aims*, the sentence “…mental health community support services at transfer of care for those with mental health concerns or who are in suicidal crisis?” was changed to “…mental health community support services for people with mental health concerns or who are in suicidal crisis?”
- Under *Research Questions*, in the first paragraph, the phrase “with those with lived experience” was changed to “with people with lived experience.”
- Under *Research Questions*, the first question “How do those with lived experience, carers, and families experience service integration and coordination across emergency care and their NDIS providers? Are there signs and/or behaviors that 1. NDIS providers should be alert to prior to clients presenting to the ED? Can awareness of these signs and/or behaviors be a catalyst to prevent an ED presentation?” was revised as “How do those with lived experience, carers, and families experience service integration and coordination across emergency care and their NDIS providers? Are there concerns and preferences that 1. NDIS providers should be alert to prior to clients presenting to the ED? Can awareness of these concerns and preferences be a catalyst to prevent an ED presentation?”
- Under *Study Design*, the sentence “The phenomena to be discovered and interpreted for this study are:…” was changed to “The study is focused on exploring and interpreting….”
- Under *Study Design*, the sentence “Data collection will first aim to discover the lived experience voice…” was changed to “Data collection will first aim to elicit the lived experience voice….”
- Under *Study Design*, the sentence “Primarily, the lived experience interviews and of NDIS support workers/coordinators (participant group 2) with interviews and of NDIS…” was changed to “Primarily, lived experience perspectives, including both consumer and carer perspectives will be generated via interviews and NDIS….”

**Table 1**

- In Table 1, under column *Exclusion criteria* and row *1*, the phrase “Anyone that is unwell and unable to give informed consent;” was changed to “Anyone that is unable to give informed consent;”

**Results**

- The first sentence “The results of this study will provide insight for strengthening discharge communication pathways to enhance continuity of care to improve connection with community mental health services and outcomes for patients” was changed to “The results of this study will provide insight for strengthening discharge communication pathways between EDs and community mental health services so that these reflect person-centered and person-led care outcomes.”

**Discussion**

- Under *Conclusion*, the sentence “…lived experience to stay well and to inform health policy” was changed to “…lived experience to have improved voice and influence health policy.”

The corrections will appear in the online version of the paper on the JMIR Publications website on November 25, 2021, together with the publication of this correction notice. Because this was made after submission to PubMed, PubMed Central, and other full-text repositories, the corrected article has also been resubmitted to those repositories.

