# Supplementary material for: National Disability Insurance Scheme and Lived Experience of People Presenting to the Emergency Department: Protocol for a Mixed Methods Study
Source: JMIR Res Protoc. 2021 Nov 4;10(11):e33268. doi: 10.2196/33268 (PMC8603173; doi:10.2196/33268)
Supplement: Multimedia Appendix 1 [file resprot_v10i11e33268_app1.docx]

| **#** | **Searches** | **Results** |
| --- | --- | --- |
| 1 | Mental Health/ | 41114 |
| 2 | exp Personality Disorders/ | 41720 |
| 3 | exp Mental Disorders/ | 1264613 |
| 4 | exp Substance-Related Disorders/ | 282766 |
| 5 | Stress Disorders, Traumatic/ | 713 |
| 6 | Psychological Distress/ | 1245 |
| 7 | exp Self-Injurious Behavior/ | 72355 |
| 8 | exp Homeless person/ | 9261 |
| 9 | exp Personality Disorder/ | 41720 |
| 10 | exp "schizophrenia spectrum and other psychotic disorders"/ | 149593 |
| 11 | exp depressive disorder/ | 110779 |
| 12 | Stress Disorders, Post-Traumatic/ | 33713 |
| 13 | (Psychosocial disab* or Psychological disorder or psychological distress or mental* ill* or Mental health* or post traumatic stress or posttraumatic stress or ptsd or personality disorder* or borderline personality disorder* or schizophren* or major depressive disorder* or schizoaffective disorder* or anxiety disorder* or bipolar or manic depress* or mood disorder* or neuros* or neurotic disorder* or depression or affective disorder* or Dissociative disorder or obsessive compulsive disorder or OCD or stress disorder* or addiction or substance abuse or drug abuse or inhalant abuse or alcohol abuse or cocaine abuse or substance induced psychosis or marijuana abuse or substance related disorder* or homeless* or suicid* or self injur* or parasuicid* or selfharm or self harm).tw,kw. | 1011163 |
| 14 | 1 or 2 or 3 or 4 or 5 or 6 or 7 or 8 or 9 or 10 or 11 or 12 or 13 | 1893198 |
| 15 | exp Emergency Service, Hospital/ | 81735 |
| 16 | Emergency Services, Psychiatric/ | 2465 |
| 17 | Crisis Intervention/ | 5692 |
| 18 | Critical Care/ | 53659 |
| 19 | After-Hours Care/ | 1901 |
| 20 | (emergency service* or emergency room or ED or emergency hospitalisation psychiatric service* or psychiatric emergency service* or ED or psychiatric admission or crisis intervention or critical care or after hours care or unscheduled health care or urgent care or crisis care or unscheduled care or unscheduled medical care).tw,kw. | 146846 |
| 21 | 15 or 16 or 17 or 18 or 19 or 20 | 230596 |
| 22 | "Referral and Consultation"/ | 67868 |
| 23 | Case Management/ | 10182 |
| 24 | Patient Discharge Summaries/ | 256 |
| 25 | "Continuity of Patient Care"/ | 19493 |
| 26 | intersectoral collaboration/ | 2195 |
| 27 | (intersectoral collaboration or documentation or electronic health record or referral or patient discharge summar* or consultation or case management or handover or handoff* or continuity of patient care or delivery of health care or continuity of care or treatment plan* or pathway* or transfer of care or patient discharge* or user experience*).tw,kw. | 1478418 |
| 28 | ((transition or transfer) adj5 communication).tw,kw. | 1238 |
| 29 | 22 or 23 or 24 or 25 or 26 or 27 or 28 | 1537557 |
| 30 | 14 and 21 and 29 | 3516 |
| 31 | limit 30 to yr="2010 -Current" | 1872 |
