# Supplementary figures and images for: National Disability Insurance Scheme and Lived Experience of People Presenting to the Emergency Department: Protocol for a Mixed Methods Study
Source: JMIR Res Protoc. 2021 Nov 4;10(11):e33268. doi: 10.2196/33268 (PMC8603173; doi:10.2196/33268)

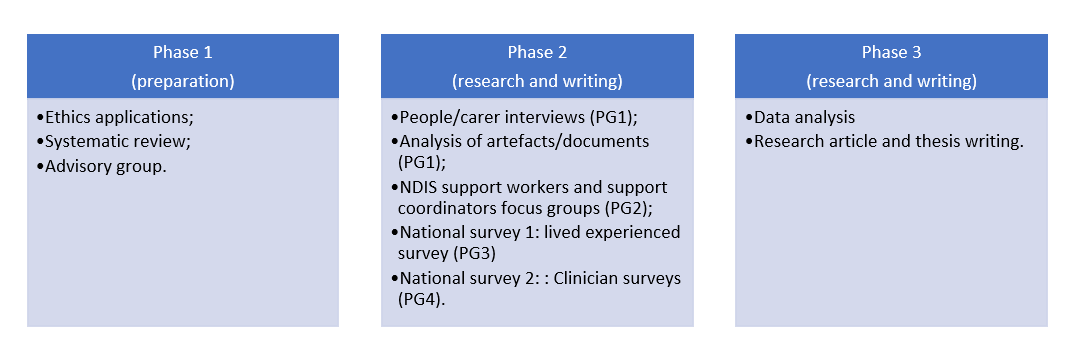

Supplement: Multimedia Appendix 2 [file resprot_v10i11e33268_app2.png]
