# Supplementary material for: National Disability Insurance Scheme and Lived Experience of People Presenting to the Emergency Department: Protocol for a Mixed Methods Study
Source: JMIR Res Protoc. 2021 Nov 4;10(11):e33268. doi: 10.2196/33268 (PMC8603173; doi:10.2196/33268)
Supplement: Multimedia Appendix 3 [file resprot_v10i11e33268_app3.pdf]

Clinical & Health Sciences

Panel Assessment Form

**Name of Candidate**	**Heather McIntyre**
**Name of Panel Member**	**Dr Melissa Petrakis**

My opinion of the student's proposal is summarised below *[Tick where appropriate]*

**Topic**	**Satisfactory**	**Unsatisfactory**
The topic contributes new knowledge to the subject area	✓	
The topic has sufficient scope for doctoral research	✓	
The proposed research has a sound policy, philosophical, scientific or theoretical base	✓	
The proposed application of the research to issues in health and health care is discussed	✓	
To my knowledge, the material in this proposed research has not been published before	✓	

**Literature Review**	**Satisfactory**	**Unsatisfactory**
Succinct summary of the relevant literature was provided in the proposal	✓	
The literature cited by the student is based chiefly on primary sources	✓	
The sources of evidence on which the proposed research is based are clearly stated	✓	
The interpretations and conclusions are justified by the evidence presented	✓	

**Proposal**	**Satisfactory**	**Unsatisfactory**
The title of the research accurately reflects the content of the proposal	✓	
Clear and logical description of proposed research is presented	✓	
The writing style is grammatically correct, and references are cited appropriately and correspond accurately to the conventions used by the Academic Unit	✓	
A 300-word abstract is included in the proposal that accurately reflects the proposed research	✓	
The research proposal does not exceed 20 pages	✓	
Sufficient detail of proposed research is provided to allow evaluation	✓	
The project appears feasible, given the support, timelines and resources available to the student	✓	

Clinical & Health Sciences

Research Method	Satisfactory	Unsatisfactory
There is a clear statement of the purpose, aim, question or hypothesis of the research		✓
All key concepts are clearly defined (as appropriate)	✓	
Study design is clearly described. *eg: participant criteria, recruitment and sample size justification, or animal model used.*		✓
Method is clearly described. *eg: what data will be collected, how the data will be collected and the processes that will be used to ensure accuracy of data. Includes any data collection tools, scales or instruments that will be used.*	✓	
The proposed data analysis is clearly described, justified and appropriate		✓
The ethical aspects of the research are addressed (as appropriate)		✓
The ethical approval and any other permissions that are required to conduct research are described	✓	
Any resource implications of the proposed research are adequately addressed	✓	

Are there any cost implications for this proposed study? Yes ✓ No ☐

If yes, have these costs been addressed in the proposal? Yes ✓ No ☐

Have you identified any potential project risks? Yes ☐ No ✓

(This could be in terms of legal, ethical or technical matters and beyond the normal timeline and resource-related risks to a project. Examples may include: use of sensitive, intimate data about people and concerns about its storage; work involving vulnerable populations including children; use of very dangerous equipment or reagents.)

Please provide any comments in the Review Feedback section below.

Reviewer feedback to candidate

This is a very interesting and potentially worthwhile project.

The proposal has been well thought out and well written.

The 3 phases make sense and provide an appropriate structure and timeline for a PhD project.

The candidate and her supervisors are to be commended for tackling a high needs group, a crisis setting that is volatile and multi-faceted and complex, and an approach that is (logically) exploratory and (usefully) interested in a lived experience perspective in the (evolving) NDIS.

The issues with this application are resolvable and likely represent the stage of the candidate's journey. At present though there are major issues with incongruity. They render the title inaccurate: this is unlikely to capture '***lived experience of psychosocial disability'*** and is not a 'co-enquiry study'.

For a study professing to be interested in and designed to engage in co-design, the research questions are too well defined without a process yet of engagement with lived experience.

In fact even whether *any* consumers and carers have had input into the design of the study to date is not clear, and it needs to be transparent (and present), i.e. page 10:

> **2.1 Research questions**
> These are the emerging research questions determined from consultations with MIND Australia staff, the research team and others in the sector:
> 1. How do those with lived experience, carers and families experience service integration and coordination across emergency care and their NDIS providers? Are there signs and/or behaviours that NDIS providers should be alert to prior to clients' presenting to the emergency department? Can awareness of these signs and/or behaviours be a catalyst to prevent an emergency department presentation?
> 2. What are the barriers to accessing therapeutic treatment within the emergency department through the health/disability/mental health interface (NDIS services and emergency departments) and how can these be transcended for improved person-centred care and recovery?
> 3. How do emergency care clinicians connect with network of NDIS providers in terms of coordination of information, support, and involvement in assessment, treatment planning and transfer of care? What works well? What doesn't work well? What could be done better?
> These questions are based on consultation, reporting, gaps in the literature, concerns about the use of isolation and restraint, and reports of current practices within the emergency department.

As a result of the design being researcher/staff-led so too the research questions represent researcher/staff wording and preoccupations. This is very odd in a study that aims p. 10 'Firstly, the aim is to discover the lived experience voice of those with a psychosocial disability and carers'.

Recruitment (p. 13): That there are only to be 14 consumers and 7 carers, yet about 20 support workers…and some 100 clinicians! Really? The 'co' is barely there (potentially) in this enquiry.

These research questions, as worded, will not establish a study that will invite participants to share their experiences in using an emergency department. The approach would need to be interested in things such as: *why did they accessed it? When? What did they hope for? Were their wants met? Were their needs met? Who did they hope would help them? How? For how long? Doing what? In which space? With which tools or equipment? Did that happen? Did they feel safe? Listened to? Helped? What helped the most? How could they tell people were helpful? What should workers in EDs do more of? Less of?*

What reasons did they have for saying what was good? or could be changed to be better?

I hope this helps with some rethinking of the approach. Or acknowledgement of researcher/worker focus.

Please include additional typed information, comments and critique of the proposal that can be sent to the candidate and supervisors.

Reviewer signature: _______ Melissa Petrakis _________ Date: ________13/12/20____
