# Supplementary material for: National Disability Insurance Scheme and Lived Experience of People Presenting to the Emergency Department: Protocol for a Mixed Methods Study
Source: JMIR Res Protoc. 2021 Nov 4;10(11):e33268. doi: 10.2196/33268 (PMC8603173; doi:10.2196/33268)
Supplement: Multimedia Appendix 4 [file resprot_v10i11e33268_app4.docx]

***Panel Assessment Form***

| **Name of Candidate** | **Heather McIntyre** |
| --- | --- |
| **Name of Panel Member** | **Dr Peter ‘Kevin’ O’Shaughnessy** |

My opinion of the student’s proposal is summarised below *[Tick where appropriate]*

| **Topic** | **Satisfactory** | **Unsatisfactory** |
| --- | --- | --- |
| The topic contributes new knowledge to the subject area |  |  |
| The topic has sufficient scope for doctoral research |  |  |
| The proposed research has a sound policy, philosophical, scientific or theoretical base |  |  |
| The proposed application of the research to issues in health and health care is discussed |  |  |
| To my knowledge, the material in this proposed research has not been published before |  |  |

| **Literature Review** | **Satisfactory** | **Unsatisfactory** |
| --- | --- | --- |
| Succinct summary of the relevant literature was provided in the proposal |  |  |
| The literature cited by the student is based chiefly on primary sources |  |  |
| The sources of evidence on which the proposed research is based are clearly stated |  |  |
| The interpretations and conclusions are justified by the evidence presented |  |  |

| **Proposal** | **Satisfactory** | **Unsatisfactory** |
| --- | --- | --- |
| The title of the research accurately reflects the content of the proposal |  |  |
| Clear and logical description of proposed research is presented |  |  |
| The writing style is grammatically correct, and references are cited appropriately and correspond accurately to the conventions used by the Academic Unit |  |  |
| A 300-word abstract is included in the proposal that accurately reflects the proposed research |  |  |
| The research proposal does not exceed 20 pages |  |  |
| Sufficient detail of proposed research is provided to allow evaluation |  |  |
| The project appears feasible, given the support, timelines and resources available to the student |  |  |

| **Research Method** | **Satisfactory** | **Unsatisfactory** |
| --- | --- | --- |
| There is a clear statement of the purpose, aim, question or hypothesis of the research |  |  |
| All key concepts are clearly defined (as appropriate) |  |  |
| Study design is clearly described.  *eg: participant criteria, recruitment and sample size justification, or animal model used.* |  |  |
| Method is clearly described.  *eg: what data will be collected, how the data will be collected and the processes that will be used to ensure accuracy of data. Includes any data collection tools, scales or instruments that will be used.* |  |  |
| The proposed data analysis is clearly described, justified and appropriate |  |  |
| The ethical aspects of the research are addressed (as appropriate) |  |  |
| The ethical approval and any other permissions that are required to conduct research are described |  |  |
| Any resource implications of the proposed research are adequately addressed |  |  |

Are there any cost implications for this proposed study? Yes x No

If yes, have these costs been addressed in the proposal? Yes x No

Have you identified any potential project risks? Yes  No x

*(This could be in terms of legal, ethical or technical matters and beyond the normal timeline and resource-related risks to a project. Examples may include: use of sensitive, intimate data about people and concerns about its storage; work involving vulnerable populations including children; use of very dangerous equipment or reagents.)*

Please provide any comments in the Review Feedback section below.

**Reviewer feedback to candidate**

Thank you for the opportunity to review this proposal. Heather has chosen a very interesting and important area to research. As both an experienced Emergency Department nurse and at times carer for family members with long chronic mental health disorders this research was of great interest to me. This thesis has made it clear that there are many gaps that remain in research into how people with psychosocial disability under the NDIS can be better cared for and listened to in the Emergency Department setting.

Heather, your writing style is clear and easy to read and I found few errors in your grammar, spelling and/or referencing. You been able to provide insight into the gaps in knowledge regarding the experiences of people with psychosocial disability under the NDIS and the clinicians that care for them within the South Australian Emergency Department system.

You have established and described a clear research plan that is appropriate for both the research question and the time that you have had available to complete your research.

You should be congratulated on both completing a literature review and eliciting views of experts/people with lived experience. Many students struggle to complete this.

My comments, questions and criticisms are superficial and do not take away from your fine work.

- I would suggest you focus on the South Australian experiences of clinicians.
- Focus groups with Emergency Department clinicians may give you insights into their experiences/ barriers to care as well as providing a framework for your survey.
- It is important to note that Focus Groups lack confidentiality and it is worthwhile to have a sentence or two explaining how you would address this.

**Good Luck,**

**Kevin**

**Please include additional typed information, comments and critique of the proposal that can be sent to the candidate and supervisors.**

Reviewer signature: ____________________________________ Date: _____________________
